# Supplementary material for: Roles of Srs2/PARI-family DNA helicases in NoCut checkpoint signaling and abscission regulation
Source: J Cell Biol. 2025 Oct 31;224(12):e202502014. doi: 10.1083/jcb.202502014 (PMC12577367; doi:10.1083/jcb.202502014)
Supplement: Table S1 — shows Saccharomyces cerevisiae strains. [file jcb_202502014_tables1.docx]

**Table S1: *Saccharomyces cerevisiae* strains**

| **Strain n** | **Background** | **Genotype** |
| --- | --- | --- |
| 1335 | S288C | *MATa ADEGV:URA3 pGAL1:GFP-CAAX:HIS3 SPC42-GFP:HphMX leu2 lys2-801 ade2-101 trp1Δ63* |
| 3399 | S288C | *MATalpha srs2Δ::NAT ADEGV:URA3 pGAL1:GFP-CAAX:HIS3 SPC42-GFP:HphMX leu2 lys2-801 ade2-101 trp1Δ63* |
| 2378 | S288C | *MATa top2-4 ADEGV:URA3 pGAL1:GFP-CAAX:HIS3 SPC42-GFP:HphMX leu2 lys2-801 ade2-101* |
| 3401 | S288C | *MATa srs2Δ::NAT top2-4 ADEGV:URA3 pGAL1:GFP-CAAX:HIS3 SPC42-GFP:HphMX leu2 lys2-801 ade2-101* |
| 3949 | BY4741 | *MATa RFA2-GFP:HIS3 HTB2-mCherry:HphMX* |
| 6161 | BY4741 | *MATa srs2Δ::NAT RFA2-GFP:HIS3 HTB2-mCherry:HphMX* |
| 6582 | BY4741 | *MATa srs2Δ::NAT top2-4 RFA2-GFP:HIS3 HTB2-mCherry:HphMX* |
| 6583 | BY4741 | *MATa top2-4 RFA2-GFP:HIS3 HTB2-mCherry:HphMX* |
| 4581 | S288C | *MATalpha POL30-S115P ADEGV:URA3 pGAL1:GFP-CAAX:HIS3 SPC42-GFP:HMX leu2 lys2-801 ade2-101 trp1Δ63* |
| 4571 | S288C | *MATa SRS2ΔSIM::NAT top2-4 ADEGV:URA3 pGAL1:GFP-CAAX:HIS3 SPC42-GFP:HphMX leu2 lys2-801 ade2-101* |
| 3960 | S288C | *MATa SRS2ΔSIMΔPIP::NAT top2-4 ADEGV:URA3 pGAL1:GFP-CAAX:HIS3 SPC42-GFP:HphMX leu2 lys2-801 ade2-101* |
| 6574 | S288C | *MATa SRS2ΔPIP::NAT top2-4 ADEGV:URA3 pGAL1:GFP-CAAX:HIS3 SPC42-GFP:HphMX leu2 lys2-801 ade2-101* |
| 1984 | S288C | *MATa HTB2-mCherry::URA3 MYO1-GFP::HIS3 leu2 lys2-801 ade2-101 trp1Δ63* |
| 2676 | S288C | *MATa top2-4 HTB2-mCherry::URA3 MYO1-GFP::HIS3 leu2 lys2-801 ade2-101 trp1Δ63* |
| 6570 | S288C | *MATa srs2Δ::NAT HTB2-mCherry::URA3 MYO1-GFP::HIS3 leu2 lys2-801 ade2-101 trp1Δ63* |
| 6571 | S288C | *MATa srs2Δ::NAT top2-4 HTB2-mCherry::URA3 MYO1-GFP::HIS3 leu2 lys2-801 ade2-101 trp1Δ63* |
| 6572 | S288C | *MATa SRS2ΔSIM::NAT top2-4 HTB2-mCherry::URA3 MYO1-GFP::HIS3 leu2 lys2-801 ade2-101 trp1Δ63* |
| 6573 | S288C | *MATa SRS2ΔPIPΔSIM::NAT top2-4 HTB2-mCherry::URA3 MYO1-GFP::HIS3 leu2 lys2-801 ade2-101 trp1Δ63* |
| 6575 | S288C | *MATa SRS2ΔPIP::NAT top2-4 HTB2-mCherry::URA3 MYO1-GFP::HIS3 leu2 lys2-801 ade2-101 trp1Δ63* |
| 2833 | S228C | *MATa ChrXII(1059):nat:ChrIV(19.5) pGAL1:CEN4:KanMX4 Htb2-mCherry:URA pGAL1:GFP-CAAX:TRP1 ura3-52 his3Δ200 leu2 lys2-801 ade2-101 trp1Δ63* |
| 4578 | S228C | *MATa elg1Δ::HYG ChrXII(1059):nat:ChrIV(19.5) pGAL1:CEN4:KanMX4 Htb2-mCherry:URA pGAL1:GFP-CAAX:TRP1 ura3-52 his3Δ200 leu2 lys2-801 ade2-101 trp1Δ63* |
| 2553 | S228C | *MATa ipl1-321 top2-4 ADEGV:URA3 pGAL1:GFP-CAAX:HIS3 SPC42-GFP:HphMX leu2 lys2-801 ade2-101* |
| 6623 | S228C | *MATa srs2Δ::NAT ipl1-321 top2-4 ADEGV:URA3 pGAL1:GFP-CAAX:HIS3 SPC42-GFP:HphMX leu2 lys2-801 ade2-101* |
