# Supplementary material for: Roles of Srs2/PARI-family DNA helicases in NoCut checkpoint signaling and abscission regulation
Source: J Cell Biol. 2025 Oct 31;224(12):e202502014. doi: 10.1083/jcb.202502014 (PMC12577367; doi:10.1083/jcb.202502014)
Supplement: Table S2 — shows siRNA sequences for gene knockdown. [file jcb_202502014_tables2.docx]

**Table S2. siRNA sequences for gene knockdown**

| **siRNA** | **Sequence 5’-3’** |
| --- | --- |
| siCtrl pool | UAAGGCUAUGA AGAGAUAC,  AUGUAUUGGCCU-GUAUUAG,  AUGAACGUGAAUUGCUCAA,  UGGUUUACAUGUCGA-CUAA |
| siPARI-1 | GAATAGATTGTACGGCAAA |
| siPARI-2 | CCAAGGACAAGTTGATTTC |
| siNUP153 | GAGGAGAGCUCUAAUAUUA |
