# Supplementary material for: Roles of Srs2/PARI-family DNA helicases in NoCut checkpoint signaling and abscission regulation
Source: J Cell Biol. 2025 Oct 31;224(12):e202502014. doi: 10.1083/jcb.202502014 (PMC12577367; doi:10.1083/jcb.202502014)
Supplement: Table S3 — shows primer sequences for qPCR. [file jcb_202502014_tables3.docx]

**Table S3. Primer sequences for qPCR**

| **Target gene** | **Forward/Reverse** | **Sequence 5’-3’** |
| --- | --- | --- |
| ACTIN B | Forward | AGGCACCAGGGCGTGAT |
| ACTIN B | Reverse | GCCCACATAGGAATCCTTCTGAC |
| PARI | Forward | GCATCAAAGCCTTTGTGTG |
| PARI | Reverse | CCTGTCTGACTGGTTGAT |
