# Supplementary material for: Roles of Srs2/PARI-family DNA helicases in NoCut checkpoint signaling and abscission regulation
Source: J Cell Biol. 2025 Oct 31;224(12):e202502014. doi: 10.1083/jcb.202502014 (PMC12577367; doi:10.1083/jcb.202502014)
Supplement: Table S4 — shows antibodies used for western blot and immunofluorescence. [file jcb_202502014_tables4.docx]

**Table S4. Antibodies used for western blot and immunofluorescence**

| **Primary antibodies** | | | |
| --- | --- | --- | --- |
| **Raised in** | **Against** | **Reference** | **Dilution** |
| Mouse | a-Tubulin | T9026 | 1:1000 (IF, PFA) |
| Mouse | Nup153 | ab24700 | 1:1,000 (WB) |
| Rabbit | a-Tubulin | ab52866 | 1:1000 (IF, PFA)  1:5,000 (WB) |
| Rabbit | pT323 Aurora B | 600-401-677 | 1:500 (IF) |
| Mouse | MRE11 | sc-135992 | 1:500 (IF) |
| Mouse | TOP-IIα | sc-166934 | 1:200 (IF) |
| **Secondary antibodies** | | | |
| Goat | Anti-mouse IgG  Alexa Fluor Plus 488 | A32723 | 1:500 |
| Goat | Anti-mouse IgG  Alexa Fluor 568 | A-11031 | 1:500 |
| Goat | Anti-rabbit IgG  Alexa Fluor 488 | A32731 | 1:500 |
| Goat | Anti-rabbit IgG  Alexa Fluor 647 | A32733 | 1:500 |
| Goat | Anti-mouse IgG  HRP | 170-6516 | 1:10,000 |
| Goat | Anti-rabbit IgG  HRP | 31460 | 1:10,000 |
