# Supplementary figures and images for: Roles of Srs2/PARI-family DNA helicases in NoCut checkpoint signaling and abscission regulation
Source: J Cell Biol. 2025 Oct 31;224(12):e202502014. doi: 10.1083/jcb.202502014 (PMC12577367; doi:10.1083/jcb.202502014)

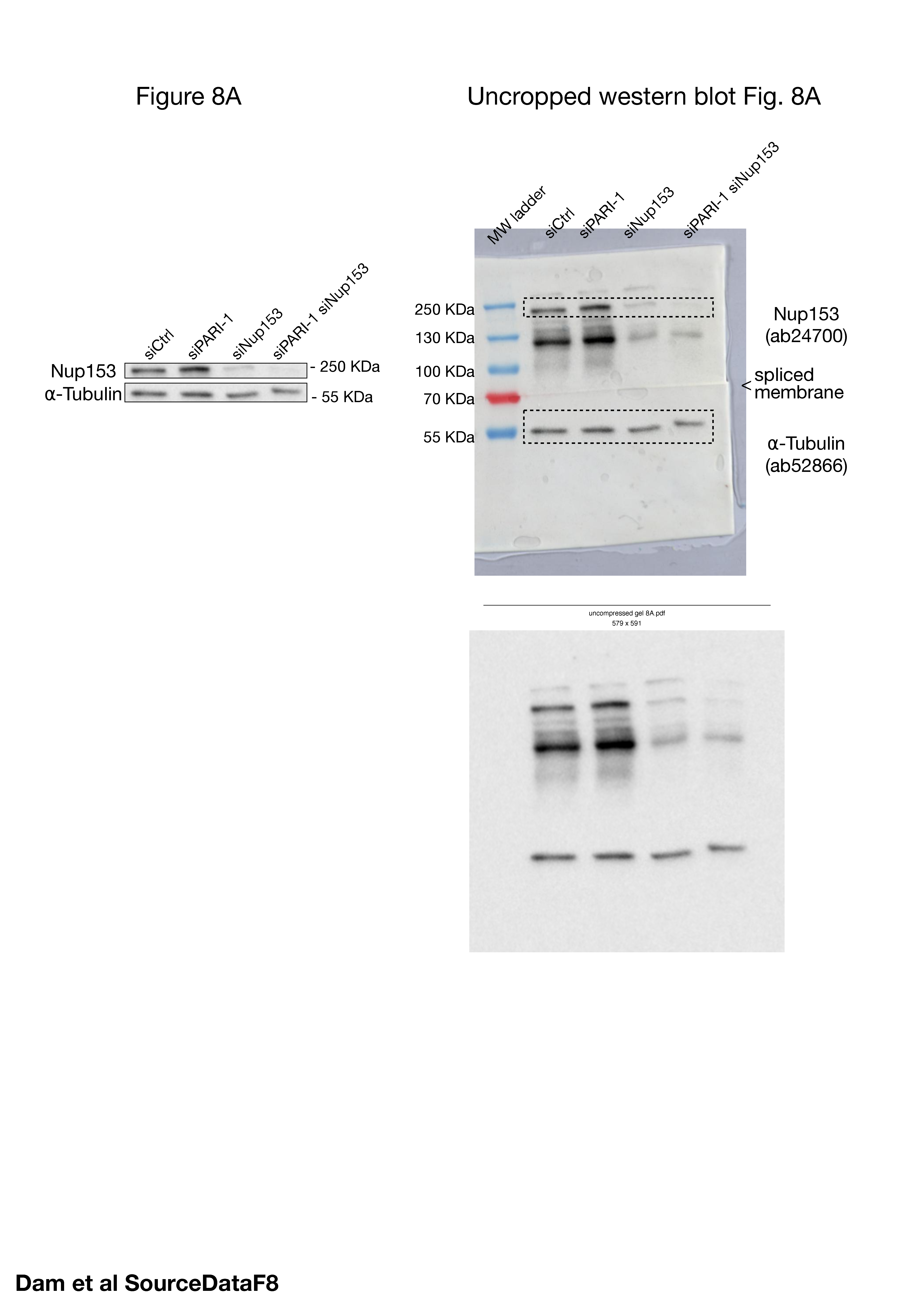

Supplement: SourceData F8 — is the source file for Fig. 8. [file jcb_202502014_sourcedataf8.png]
